# Indo-China Monsoon Indices

**DOI:** 10.1038/srep08107

**Published:** 2015-01-29

**Authors:** ChinLeong Tsai, Swadhin K. Behera, Takuji Waseda

**Affiliations:** 1 Graduate School of Frontier Sciences, The University of Tokyo, Japan; 2 Application Laboratory, JAMSTEC, Yokohama, Japan

## Abstract

Myanmar and Thailand often experience severe droughts and floods that cause irreparable damage to the socio-economy condition of both countries. In this study, the Southeastern Asian Summer Monsoon variation is found to be the main element of interannual precipitation variation of the region, more than the El Niño/Southern Oscillation (ENSO). The ENSO influence is evident only during the boreal spring season. Although the monsoon is the major factor, the existing Indian Monsoon Index (IMI) and Western North Pacific Monsoon Index (WNPMI) do not correlate well with the precipitation variation in the study regions of Southern Myanmar and Thailand. Therefore, a new set of indices is developed based on the regional monsoon variations and presented here for the first time. Precipitation variations in Southern Myanmar and Thailand differ as well as the elements affecting the precipitation variations in different seasons. So, separate indices are proposed for each season for Southern Myanmar and Thailand. Four new monsoon indices based on wind anomalies are formulated and are named as the Indochina Monsoon Indices. These new indices correlate better with the precipitation variations of the study region as compared to the existing IMI and WNPMI.

Severe floods and droughts in Southern Myanmar and Thailand have greatly damaged this region economically. For example, Thailand lost 45.7 billion USD due to a flood in 2011 alone. What is more, Thailand lost 50 million USD due to a drought in 2010. As Thailand is one of the main exporters for hard disk drive (HDD), the flood in 2011 caused an acute shortage of HDD in the global market, which shows the extent of the climate influence on the interconnected global economy in the modern world. Therefore, a forecast for flood and drought is greatly in need for the Indochina region. However, a reliable forecast system can only be built if we have clear understanding of the underlying factors causing the precipitation variations in this region.

This study analyses variations in monsoon rainfall for three main seasons: March, April and May (MAM), June, July and August (JJA), and September, October and November (SON). The analysis is stratified into three seasons because much of the annual precipitations in Southern Myanmar and Thailand occur during these three seasons. The analyses reveal that the main element causing the precipitation variation in this region is the Southeastern Asian Summer Monsoon. Since precipitation variations generally differ from west to east in the region (Fig. 1(b)), the study domain is divided into Southern Myanmar study domain and Thailand study domain (which also includes small parts of Laos and Cambodia due to the rectangular nature of the chosen domain) for further analyses. Although these two study domains are greatly affected by monsoon as suggested by some earlier studies^1^,^2^, the two existing monsoon indices, Indian Monsoon Index (IMI)^3^,^4^ and Western North Pacific Monsoon Index (WNPMI)^3^,^4^, do not correlate well with the precipitation variations of these two study domains. Therefore, a new set of monsoon indices is designed in this study to better predict such variations.

Past studies show that the 850 hPa flow pattern can be very useful in understanding the seasonal variability of Southeastern Asian Summer Monsoon^5^. Furthermore, past studies also reported that the summer monsoon in the Indo–China peninsula is strongly influenced by the local wind-terrain-precipitation interaction^6^,^7^. For example, the mountains between Myanmar and Thailand block the summer monsoon winds from the west to reach Thailand, causing more precipitation on the west side and less precipitation on the east side of the mountains. Therefore, the relationship between local wind variations and precipitation variations in this region is investigated.

Precipitation in Southern Myanmar and Thailand is considerably influenced by the Southeastern Asian Summer Monsoon, which is limited within Southeast Asia. Since the Southeastern Asian Summer Monsoon is slightly different compared to large regional monsoons such as the Indian Monsoon or the Western North Pacific Monsoon, thus IMI and WNPMI are unsuitable as monsoon indices for Southern Myanmar and Thailand. Because the precipitation in Southern Myanmar and Thailand is influenced by local monsoon winds, a new set of indices incorporating these winds was explored. To account those regional variations, a total of four monsoon indices based on local monsoon wind anomalies are developed for MAM, JJA and SON in both domains. For simplicity, these four indices are named the Indo-China Monsoon Indices (ICMIs).

This paper is organized as follows: - Factors regarding the precipitation variations in Southern Myanmar and Thailand study domains, methods for developing the indices for each season of the two domains and their correlation with the precipitation variations are discussed in “Factors Affecting Precipitation Variations in Indo-China” section. Summary and further discussion can be found in “Summary and Discussions” section. Finally, in “Methods” section, datasets and methods used in the analysis are described.

## Factors Affecting Precipitation Variations in Indo-China

### Precipitation variations and role of climate phenomena

**Figure 1:**
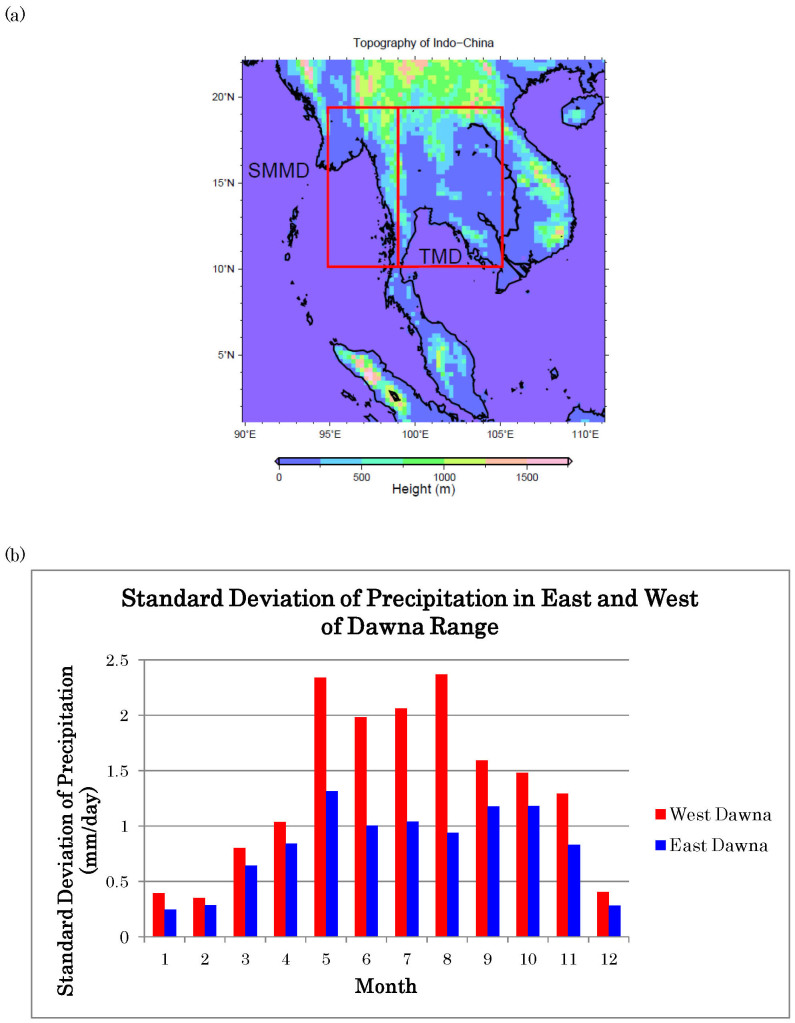
(a) Topography map (in m) for Indo-China. All the figures below except 1(b) are generated by C.L.T. using Generic Mapping Tools (GMT). (b) Monthly standard deviation of precipitation (1961–2007, in mm/day) in east side (blue bar) and west side (red bar) of Dawna Range. This figure is generated by C.L.T. using Microsoft Excel.

As there is a line of north-south orientated mountains situated on the border of Myanmar and Thailand, precipitation variations are expected to be vary between east side and west side of Dawna Range (Fig 1(a)). So, monthly standard deviation of precipitation for east side and west side of Dawna Range from 1961 to 2007 is calculated in order to clarify the variations of precipitation in these two domains (Fig 1(b)). Based on the results, the precipitation varies spatially between east and west of Dawna Range, associated with the varying impact factors. Therefore, east and west of Dawna Range are separated here into two domains, Southern Myanmar Monsoon Domain (hereafter SMMD) and Thailand Monsoon Domain (hereafter TMD), which also include small parts of Laos and Cambodia as the domain has a rectangular shape (Fig. 1(a)). Northern Myanmar is not included in this study because the precipitation variations in that domain are somewhat different from that of the southern counterpart. For the convenience of comparisons, domain means of the precipitation anomalies in both domains are calculated and later referred to as the Southern Myanmar Index (precipitation anomalies in the domain SMMD) and Thailand Index (precipitation anomalies in the domain TMD).

**Figure 2:**
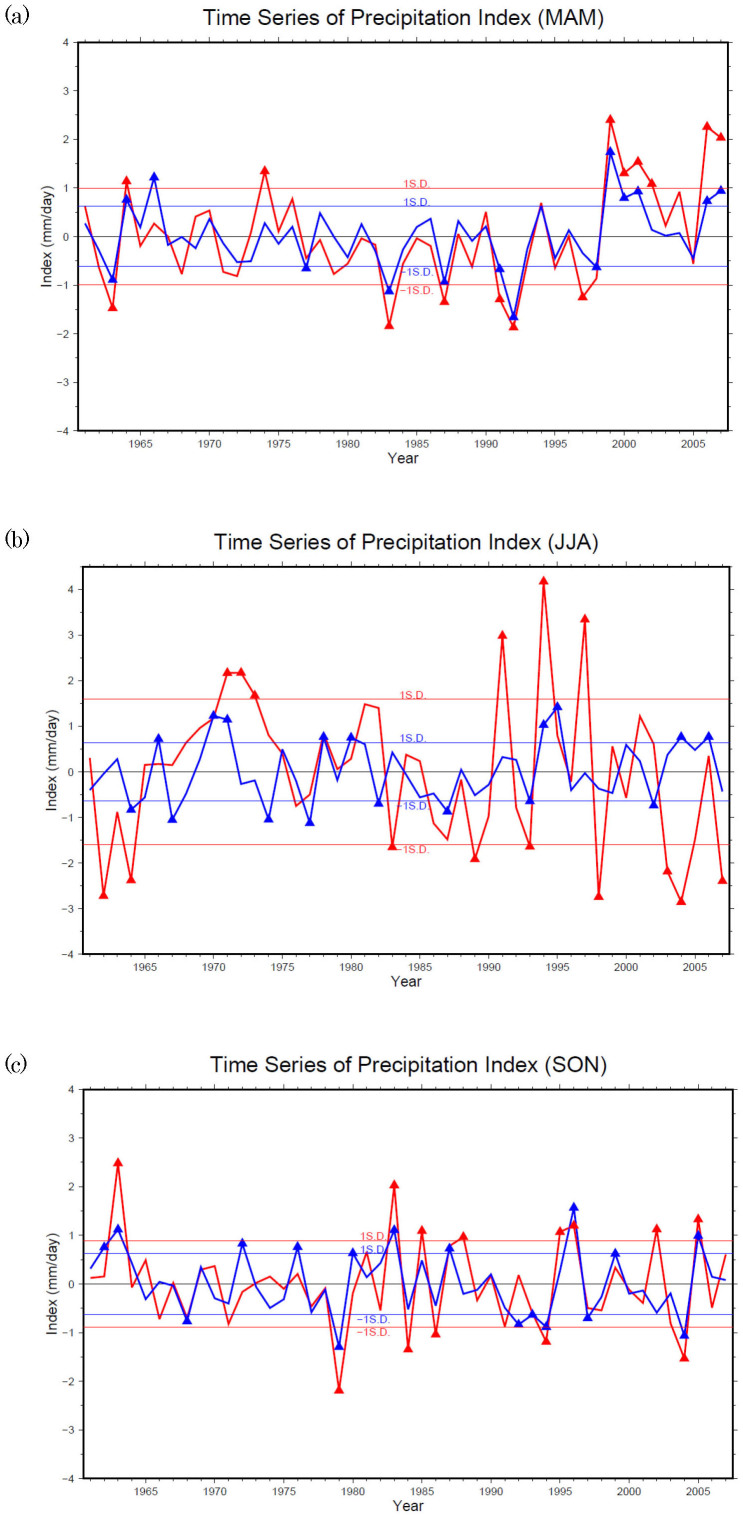
(a) Time series of the SMI (red line, mm/day) and the TI (blue line, in mm/day) during MAM of 1961–2007. Triangles are the extreme events that were selected to conduct composite analysis later. (b) Same as in 2(a), but for JJA. (c) Same as in 2(a), but for SON.

The time series of Southern Myanmar Index (SMI, red) and of the Thailand Index (TI, blue) during MAM, JJA, and SON from 1961 to 2007 are shown in Figure 2(a), (b), and (c) respectively. A comparison of the two time series reveals that the variation of both indices differs little during MAM, differs somewhat during SON but differs greatly during JJA. The correlation coefficients between them are 0.85 (MAM), 0.26 (JJA) and 0.69 (SON) respectively. The significant value for 95% confidence level by two-tailed Student t-test is 0.29. Therefore, the correlation coefficients for MAM and SON are significant, whereas the correlation coefficient for JJA is insignificant. This suggests that domain SMMD and TMD are influenced by the same factors during MAM, but these factors vary slightly during SON and greatly during JJA.

The correlation (values without brackets in Table 1) and partial correlation (by removing one's relationship among three variables) of SMI and TI with El Niño/Southern Oscillation index (Niño 3), ENSO Modoki index (EMI)^8^, and Indian Ocean Dipole Mode index (DMI)^9^ are calculated. Based on these results, we find that only Niño 3 during MAM has a partial correlation less than −0.5, which is −0.52 for SMI, and −0.63 for TI. This suggests that ENSO affects SMI and TI directly only during MAM.

### Precipitation variations and monsoon

After identifying that tropical climate phenomena are not the main factors influencing SMI and TI during JJA and SON, further analyses were conducted by making composites of extreme SMMD and TMD precipitation seasons to identify the main element causing the precipitation variations. The extreme events are picked when the index value exceeded the value of 1 standard deviation for both SMI and TI respectively. Based on this criterion for SMMD, we have identified eight (six) cases of extreme floods (droughts) during MAM, six (nine) cases of floods (droughts) during JJA, and eight (five) cases of floods (droughts) during SON. Similarly, TMD had six (seven) cases of floods (droughts) during MAM, six (four) cases of floods (droughts) during JJA after removing the IOD years, and ten (seven) cases of floods (droughts) during SON. The number of IOD events captured by the criteria was very few but those added superfluous noise to the composites during JJA because of the nonlinearity in the impacts of positive and negative phases of IOD. Composite plots are drawn using these extreme seasons. In the seasons when the wind patterns are symmetric between floods and droughts, only composite plots of flood cases are shown. But in case the winds are asymmetric for a season, both flood and drought composite plots are shown for the clarity of the discussion between the two extremities. Symmetric here means that the wind anomalies are generally mirror image to each other between flood and drought cases in a particular domain, while asymmetric means that the wind anomalies are not exactly opposite of each other between flood and drought cases.

**Figure 3:**
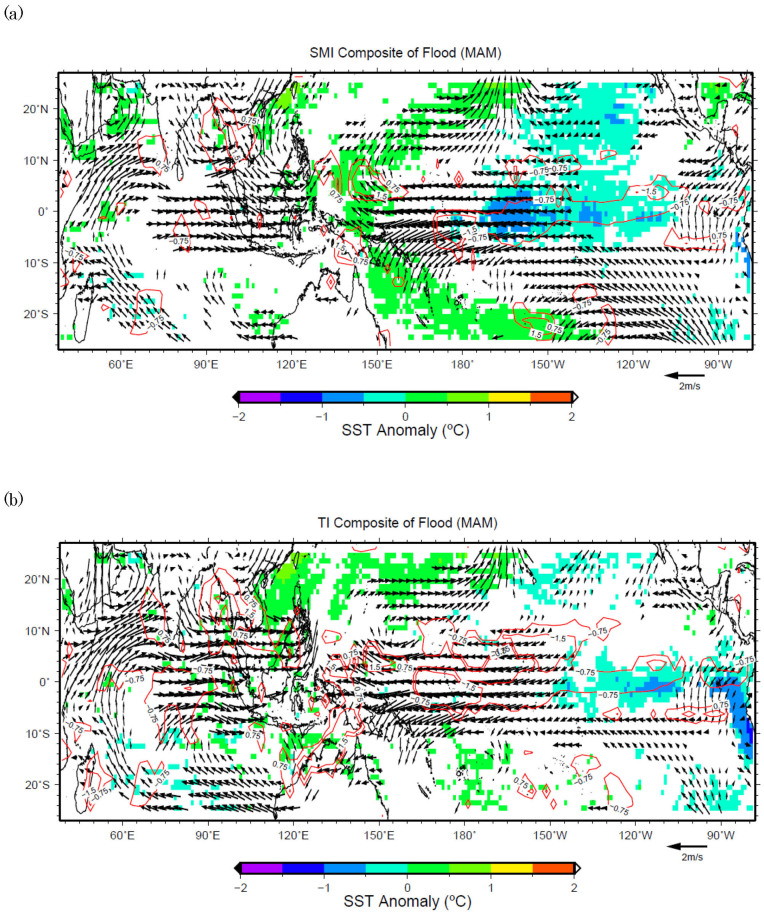
(a) Composite analysis of the flood years (years that have precipitation more than 1S.D.) during MAM in domain SMMD, the color fillings indicate SST anomalies, the vectors are the 850 hPa wind flow anomalies, and the contours are GPCP anomalies (mm/day). The color fillings and contours shown are significant values that exceed 90% two-tailed Student t-test, whereas the vectors shown are significant values with either zonal or meridional component exceed 90% two-tailed Student t-test. (b) Same as in 3(a), but for the flood years in domain TMD.

The composite analysis clarifies the role of Southeastern Asian Summer Monsoon^5^ in the rainfall variability over SMMD and TMD. However, this role differs between the domains and also among the seasons. For MAM, in addition to the influence of ENSO, the 850 hPa monsoon wind east of Sri Lanka is found to be another factor influencing SMI and TI. Because the wind patterns are symmetric between flood and drought cases, only composites of the flood cases for SMMD and TMD are shown in Fig. 3(a) and 3(b). Based on those plots, it can be deduced that the strengthening of the 850 hPa zonal wind east of Sri Lanka may bring more precipitation to SMMD and TMD. On the contrary, the weakening of the 850 hPa zonal wind east of Sri Lanka may bring less precipitation to SMMD and TMD as those winds in drought events are opposite to that in floods.

**Figure 4:**
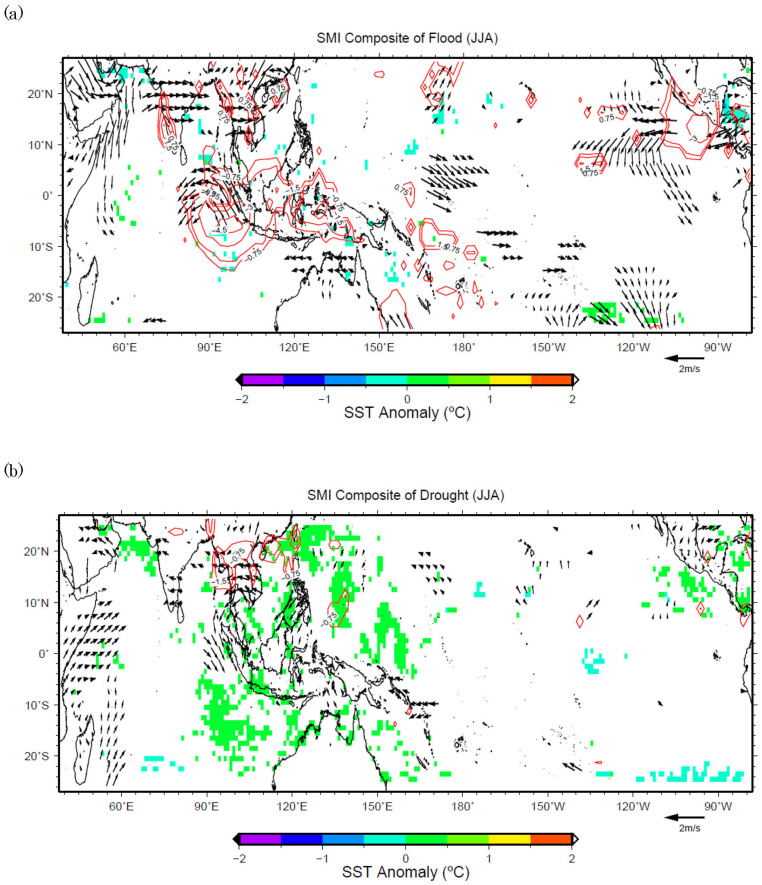
(a) Same as in Fig. 3(a), but for the flood years during JJA in domain SMMD. (b) Same as in 4(a), but for the drought years.

**Figure 5:**
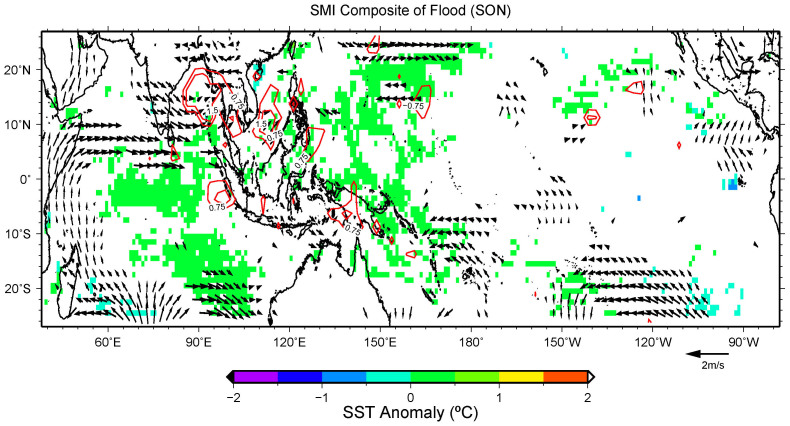
Same as in Fig.3(a), but for the flood years during SON in domain SMMD.

For SMMD during JJA, however, the patterns are more asymmetric. The strengthening of both the 850 hPa westerlies in the northern part of India and the southerlies west of Sumatra bring extra moisture to this region causing more precipitation (Fig. 4(a)). On the other hand, the weakening of the 850 hPa wind north of Sumatra may cause less precipitation in SMMD (Fig. 4(b)). During SON, the precipitation of SMMD is influenced by monsoon transition wind east of Sri Lanka (Fig. 5), that is same as MAM season. Fig. 5 shows that the strengthening of the westerlies east of Sri Lanka causes more precipitation in SMMD, and the wind patterns for drought cases are similar to those of flood years. This means the transition phase and the associated conditions of the Southeastern Asian Summer Monsoon play a major role for the precipitation in SMMD during SON season.

**Figure 6:**
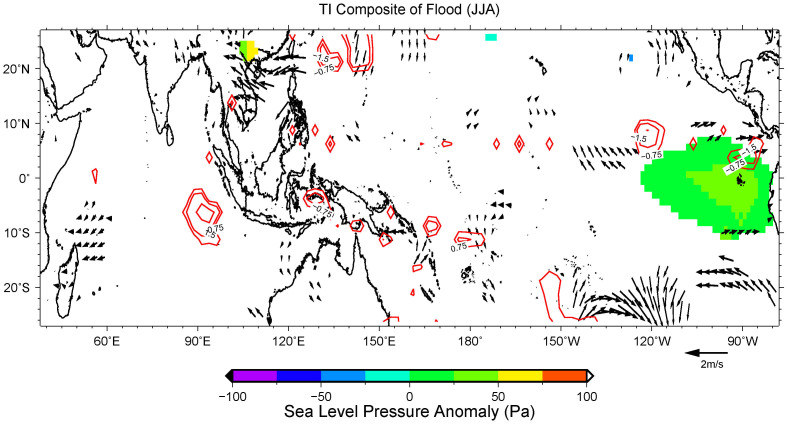
Composite analysis of the flood years during JJA in domain TMD. The color fillings indicate SLP anomalies, the vectors are the 850 hPa wind flow anomalies, and the contours are GPCP anomalies (mm/day). The color fillings and contours shown are significant values that exceed 90% two-tailed Student t-test, whereas the vectors shown are significant values with either zonal or meridional component exceed 90% two-tailed Student t-test.

**Figure 7:**
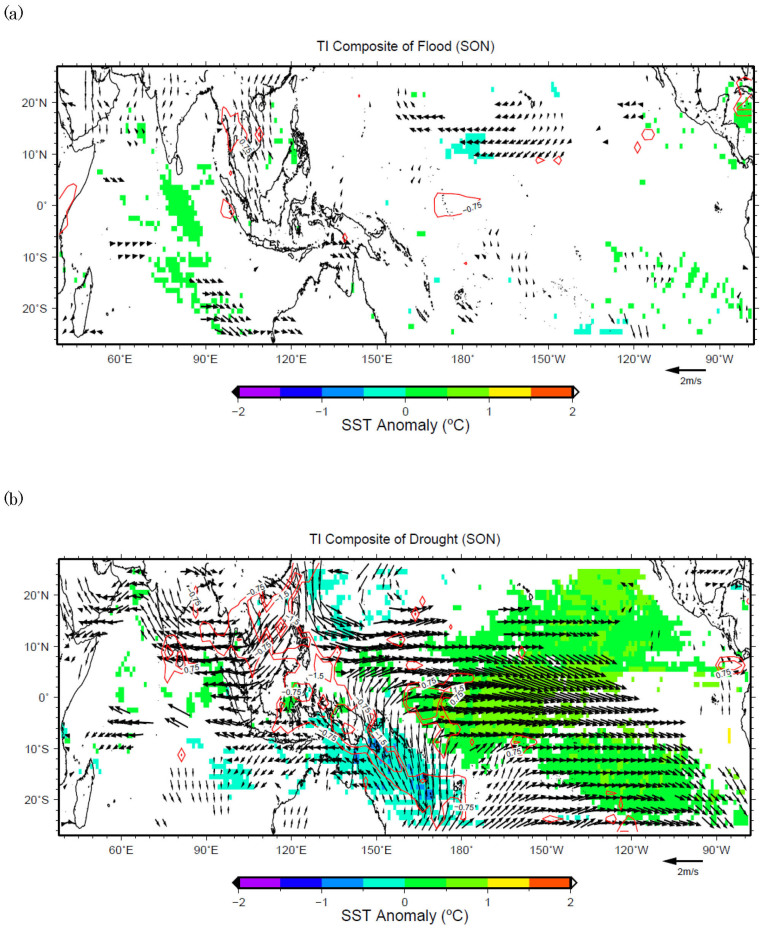
(a) Same as in Fig. 3(a), but for the flood years during SON in domain TMD. (b) Same as in 7(a), but for the drought years.

For TMD during JJA, the strengthening of the easterlies east of Hainan Island causes higher than normal precipitation (Fig. 6) in that domain. It may be noted here that the 850 hPa wind patterns are symmetric between flood and drought cases. This means that the weakening of the easterlies east of Hainan Island causes less precipitation in TMD. However, this situation changes during SON, when the wind patterns become asymmetric between flood and drought cases. Strengthening of the southerlies south of Vietnam will bring more precipitation to TMD (Fig. 7(a)), whereas weakening of the westerlies will cause less precipitation (Fig. 7(b)). This means that fast transition of Southeastern Asian Summer Monsoon and the absence of southerlies from equatorial region will cause drought, whereas normal/slow transition with the strengthening of southerlies from the equatorial region will most likely cause floods there.

### Constructing ICMI and comparing them with IMI and WNPMI

The composite analyses clearly show that SMI and TI are influenced by the designated regions of monsoon winds that carry moistures to land. These moistures will cause atmospheric instability over land when the surface air will rise through the moist adiabatic process. The rising air will trigger condensation and cloud formation, and then cause rainfall. Furthermore, these monsoon winds are different from the monsoon winds associated with that of the Indian Monsoon. Therefore, based on monsoon winds that determine the moisture flow to the two study domains, a set of indicators are formulated here to link them to the precipitation variations and possible predictions of those variations. For SMMD, during MAM and SON, the indicators are the westerlies east of Sri Lanka, represented by the 850 hPa zonal wind anomalies in the domain of 85°E–95°E, 7.5°N–12.5°N and constructed as the Indo-China Monsoon Indices Type 1 (ICMI I). The ICMIs (Supplementary Fig. 1) are the domain averages of the zonal, or zonal and meridional wind anomalies of a chosen domain. For SMMD during JJA, since SMI is affected by westerlies from India and southerlies from Sumatra, 72.5°E–85°E, 16.25°N–20°N is chosen as the domain of 850 hPa zonal wind anomalies, and 92.5°E–98.75°E, 2.5°S–10°N is chosen as the domain of 850 hPa meridional wind anomalies. The sum of the domain averages of the zonal wind anomalies and meridional wind anomalies is the ICMI of domain SMMD during JJA, which is called as Indo-China Monsoon Indices Type 2 (ICMI II).

**Figure 8:**
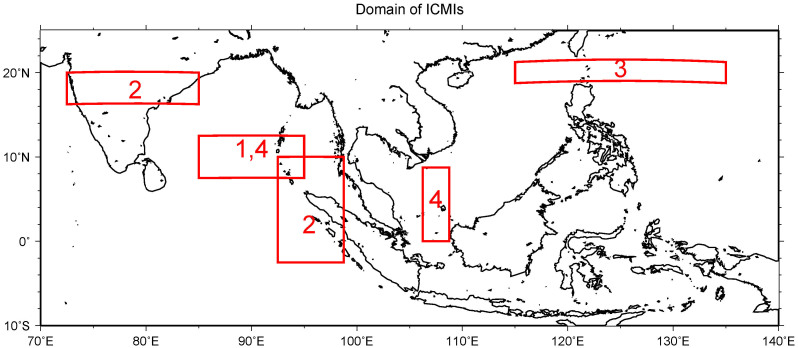
Domain of ICMIs. The boxes represent the domains of ICMIs and the numbers represent the type of ICMIs.

Since the TMD variation is same as that of the SMMD during MAM, the same monsoon index is applicable to the latter during that season. During JJA, however, TMD is affected by the easterlies east of Hainan Island. So, the domain average of 850 hPa zonal wind anomalies in the domain of 115°E–135°E, 18.75°N–21.25°N are chosen as the ICMI for TMD during JJA, which is Indo-China Monsoon Indices Type 3 (ICMI III; inverted in the analysis). During SON, TMD is affected by both the westerlies east of Sri Lanka and southerlies south of Vietnam. Hence, the domain of 85°E–95°E, 7.5°N–12.5°N is chosen for the 850 hPa zonal wind anomalies and the domain of 106.25°E–108.75°E, 0°–8.75°N is chosen for the 850 hPa meridional wind anomalies. Both domain averaged wind anomalies are also used as the Indo-China Monsoon Indices Type 4 (ICMI IV) for TMD during SON. The domains of all those ICMIs are shown in Figure 8.

Partial correlation between ICMIs, IMI, and WNPMI with SMI and TI is calculated for the comparison between ICMIs, IMI, and WNPMI (Table 1). The partial correlation coefficients here are the correlation coefficients that have excluded the influences of Niño 3, EMI and DMI. The partial correlation coefficients between ICMIs with TI and SMI are all equal or above 0.5. Furthermore, the partial correlation coefficients between ICMIs with TI and SMI are higher than those with IMI and WNPMI. This means that ICMIs are more accurate than IMI and WNPMI for indicating the strength of Southeastern Asian Summer Monsoon.

## Summary and Discussions

By using observational data, we discovered that precipitation variations in east and west of Dawna Range differ. Therefore, we separate them into two domains, Southern Myanmar Monsoon Domain i.e. SMMD and Thailand Monsoon Domain i.e. TMD in this study. We use the domain average of the precipitation anomalies in SMMD (as SMI), and TMD (as TI) to conduct our study. From the time series, we know that SMI and TI have almost the same variation patterns during MAM, slightly different during SON, but greatly different during JJA.

The analysis of the indices revealed that the precipitations in South Myanmar and Thailand are not related to the diabatic heating anomalies in the tropics through a Matsuno-Gill response that describes the teleconnection between the tropical heat anomalies with sub-tropical circulation (through Rossby wave response). The results of partial correlation analysis also show that SMI and TI are little influenced by tropical climate phenomena such as ENSO, ENSO Modoki and IOD, except for MAM season when SMI and TI both have partial correlation coefficients around −0.5 with ENSO. On the other hand, from the correlation and composite analyses, it is found that the variation in the large-scale monsoon influences the local monsoon wind and thereby the rainfall anomalies over SMMD and TMD through anomalous transports of moisture. As the figures have shown, the associated wind anomalies are often away, such as in places like India, Sri Lanka, and eastern Indian Ocean, from the precipitation anomalies over the study region. The regional monsoon here is connected to the Indian Monsoon and the Western North Pacific Monsoon through the large-scale processes.

The composite analyses clearly show that SMMD and TMD during MAM are influenced by the developing phase of the Southeastern Asian Summer Monsoon. This monsoon early signal can be detected from the changing zonal wind east of Sri Lanka. SMMD during JJA is influenced by the strengthening of westerlies in the northern part of India and southerlies west of Sumatra. This means that the strengthening of the two monsoon systems can trigger a flood in that domain, while weakening of monsoon near Sumatra may trigger drought there. For domain TMD during JJA, the strengthening of the easterlies east of Hainan Island brings extra precipitation to TMD and vice versa. The monsoon that hits SMMD during JJA does not affect TMD because they are mostly westerlies, and those winds are blocked by the line of north-south oriented mountains situated at the border of SMMD. Therefore, SMMD receives more precipitation but not TMD. During SON, the transition phase of the Southeastern Asian Summer Monsoon plays a major role for both domains. Slow/normal decaying Southeastern Asian Summer Monsoon will bring extra precipitation to SMMD. Moreover, normal/slow decaying Southeastern Asian Summer Monsoon and southerlies south of Vietnam are necessary to bring extra precipitation to TMD.

Based on a series of objective analyses consisting of partial regression analysis, partial correlation analysis, and composite analysis, the domains of the monsoon wind that cause the variations in SMI and TI are identified. Based on that, we selected the domains to construct ICMIs. By partial correlation analysis, we show that ICMI indices better explain the variations in SMI and TI as compared to the traditional indices of IMI and WNPMI. What is more, ICMIs have partial correlation coefficient equal or above 0.5 for all the combinations. This means that the set of ICMI is a better indicator for the monsoon for the two domains, and thus determining the factors that influence ICMI may lead to better precipitation predictions in SMMD and TMD.

The ICMIs are in general strong indicators of extreme precipitation events. However, seasonal mean ICMI III could not pick the 2011 extreme flood events in Thailand. From the analyses of that year data, it is found that JJA 2011 was actually a normal year in terms of monsoon rainfall with only two precipitation event exceeding 16 mm/day from 1^st^ June to 31^st^ August. On the other hand, a strong monsoon year like JJA 2001 had eight events exceeding 16 mm/day in the same period. We selected 16 mm/day for the detection of the event because this value is 2.5 times greater than the monthly climatology value of JJA precipitation. As ICMI III is a monsoon index, it detects extreme precipitation signals associated with an extreme monsoon event such as the JJA of 2001 but not the signal associated with a strong synoptic event in the JJA of 2011. Nevertheless, the September value of ICMI III was 2.816 that is 1.43 times of the standard deviation. We suggest that heavy monsoon precipitation in September of 2011 after the normal monsoon season of JJA would have charged the catchment area of the affected region preconditioning the extreme flood event when the typhoon hit Thailand in the beginning of October.

## Methods

### Data

We use Asian Precipitation – Highly Resolved Observational Data Integration Towards Evaluation of the Water Resources (APHRODITE) V1101R2^10^ (http://www.chikyu.ac.jp/precip/) as our primary precipitation data. Resolution of this data is 0.25° × 0.25° and is available daily for 1961 to 2007 for the study domain of 90°E–130°E, 15°S–25°N. Monthly means are calculated from daily precipitation data. We also used the Global Precipitation Climatology Project (GPCP) Version-2 Monthly Precipitation Analysis^11^ as our secondary precipitation data because APHRODITE does not contain data east of 150°E and south of 15°S. The horizontal resolution of GPCP is 2.5° × 2.5°. Since GPCP starts from 1979, the analysis period for GPCP is from 1979 to 2007, and the domain of analysis is 40°E–80°W, 25°S–25°N.

Furthermore, we use the monthly 850 hPa wind velocity data and monthly sea level pressure (SLP) data from The Japanese 55-year Reanalysis^12^ (JRA-55) provided by Japan Meteorological Agency (JMA). The horizontal resolution is 1.25° × 1.25°, the domain is the same as GPCP; the analysis period is the same as that of APHRODITE. Moreover, we also used the monthly sea surface temperature (SST) data from the HadISST^13^ provided by the UK Met Office Hadley Center. The resolution of the data is 1° × 1°; the domain and period of analysis is the same as JRA-55. Finally, to study the relationship between precipitation and climate phenomena, we use Niño 3 index (www.data.jma.go.jp/gmd/cpd/db/elnino/index/nino3idx.html) provided by Japan Meteorological Agency, El Niño Modoki Index (EMI) (www.jamstec.go.jp/frsgc/research/d1/iod/DATA/emi.monthly.txt) and Dipole Mode Index (DMI) (www.jamstec.go.jp/frcgc/research/d1/iod/DATA/dmi_HadISST_jan1958-dec2012.txt) provided by the Japan Agency for Marine-Earth Science and Technology (JAMSTEC). We use the ETOPO1 Global Relief Model data (www.ngdc.noaa.gov/mgg/global/global.html) provided by National Geophysical Data Center to construct the topography map.

To conduct our study, we first calculate the anomaly of each field by subtracting them from climatology. Then, we seasonally stratified them into 3 seasons (MAM, JJA and SON) and calculate the seasonal anomalies. Composite analyses are conducted to understand the elements affecting the precipitation variations. Finally, correlation and partial correlation analysis are conducted for comparison among IMI, WNPMI and ICMIs, and to understand the role of climate phenomena to the precipitation variations over the study domain.

## Author Contributions

C.L.T. conducted the analysis. C.L.T., S.K.B. and T.W. wrote the manuscript together.

## Supplementary Material

Supplementary Information Supplementary Figures

## Figures and Tables

**Table 1:** Correlation coefficients and partial correlation coefficients (in brackets) between SMI and TI with IMI, WNPMI, ICMIs, Niño 3 index, EMI and DMI for MAM, JJA and SON. It should be noted that partial correlation between Niño 3 with SMI and TI has excluded the influence of EMI and DMI, and the same process was done to EMI and DMI. For the partial correlation between IMI with SMI and TI, it has excluded the influence of Niño 3, EMI and DMI. The same was done for WNPMI and ICMIs. The significant value for 95% two-tailed Student t-test is 0.29

	SMI			TI		
	MAM	JJA	SON	MAM	JJA	SON
Niño 3.0	−0.51	0.16	−0.15	−0.62	−0.17	−0.05
	(−0.52)	(0.03)	(−0.13)	(−0.63)	(−0.21)	(−0.03)
EMI	−0.15	0.15	−0.14	−0.09	0.08	−0.12
	(−0.23)	(0.18)	(−0.11)	(−0.18)	(0.08)	(−0.11)
DMI	0.19	0.29	−0.05	0.11	0.03	−0.02
	(0.16)	(0.26)	(0.07)	(0.05)	(0.13)	(0.03)
IMI	0.57	0.01	0.39	0.58	0.27	0.25
	(0.48)	(0.07)	(0.42)	(0.47)	(0.20)	(0.28)
WNPMI	0.29	0.31	0.02	0.39	0.02	0.03
	(0.11)	(0.23)	(−0.04)	(0.21)	(0.02)	(0.04)
ICMI I	0.65	-	0.55	0.71	-	-
	(0.61)		(0.59)	(0.67)		
ICMI II	-	0.66	-	-	-	-
		(0.62)				
ICMI III	-	-	-	-	0.42	-
					(0.49)	
ICMI IV	-	-	-	-	-	0.58
						(0.61)

## References

[b1] Kripalani R. H., Singh S. V. & Panchawagh N. Variability of the summer monsoon rainfall over Thailand – Comparison with features over India. Int. J. Climatol. 15, 657–672 (1995).

[b2] Kripalani R. H. & Kulkarni A. Rainfall variability over South-east Asia – Connections with Indian Monsoon and ENSO extremes: New perspectives. Int. J. Climatol. 17, 1155–1168 (1997).

[b3] Wang B. & Fan Z. Choice of South Asian summer monsoon indices. Bull. Am. Meteorol. Soc. 80, 629–638 (1999).

[b4] Wang B., Wu R. & Lau K.-M. Interannual variability of Asian summer monsoon: Contrast between the Indian and western North Pacific-East Asian Monsoons. J. Clim. 14, 4073–4090 (2001).

[b5] Misra V. & DiNapoli S. The variability of the Southeast Asian summer monsoon. Int. J. Climatol. 34, 893–901 (2014).

[b6] Wang Z. & Chang C.-P. A numerical study of the interaction between the large-scale monsoon circulation and orographic precipitation over South and Southeast Asia. J. Clim. 25, 2440–2455 (2012).

[b7] Chang C.-P., Wang Z. Mcbride J. & Liu C.-H. Annual Cycle of Southeast Asia-Maritime Continent Rainfall and the Asymmetric Monsoon Transition. J. Clim. 18, 287–301 (2005).

[b8] Ashok K. *et al.* El Niño Modoki and its possible teleconnection. J. Geophys. Res., 112, C11007, 10.1029/2006JC003798 (2007).

[b9] Saji N. H., Goswami B. N., Vinayachandran P. N. & Yamagata T. A dipole mode in the tropical Indian Ocean. Nature, 401, 360-363 (1999). 16862108 10.1038/43854

[b10] Yatagai A. *et al.* APHRODITE: Constructing a long-term Daily Gridded Precipitation Dataset for Asia based on a dense network of rain gauges. Bull. Am. Meteorol. Soc. 93, 1401–1415, 10.1175/BAMS-D-11-00122.1 (2012).

[b11] Adler A. F. *et al.* The version-2 Global Precipitation Climatology Project (GPCP) Monthly Precipitation Analysis (1979-present). J. Hydrometeorol. 4, 1147–1167 (2003).

[b12] Ebita A. *et al.* The Japanese 55-year Reanalysis “JRA-55”: An interim report. SOLA 7, 149–152 (2011).

[b13] Rayner N. A. *et al.* Global analyses of sea surface temperature, sea ice, and night marine air temperature since the late nineteenth century. J. Geophys. Res. 108, 4407, 10.1029/2002JD002670 (2003).

