# Supplementary material for: Indo-China Monsoon Indices
Source: Sci Rep. 2015 Jan 29;5:8107. doi: 10.1038/srep08107 (PMC4309958; doi:10.1038/srep08107)
Supplement: Supplementary Information — Supplementary Figures [file srep08107-s1.pdf]

Indo-China Monsoon Indices Supplementary

ChinLeong Tsai[1], Swadhin K. Behera[1,2] & Takuji Waseda[1,2]

[1]*Graduate School of Frontier Sciences, The University of Tokyo, Japan*

[2]*Application Laboratory, JAMSTEC, Yokohama, Japan*

Nature Scientific Reports

December 18, 2014

Corresponding author address: ChinLeong Tsai,

Room 785 Environmental Studies Building, The University of Tokyo Kashiwa Campus

5-1-5 Kashiwanoha, Kashiwa City, Chiba Prefecture, Japan

Email: tsai@isea.k.u-tokyo.ac.jp

(a)

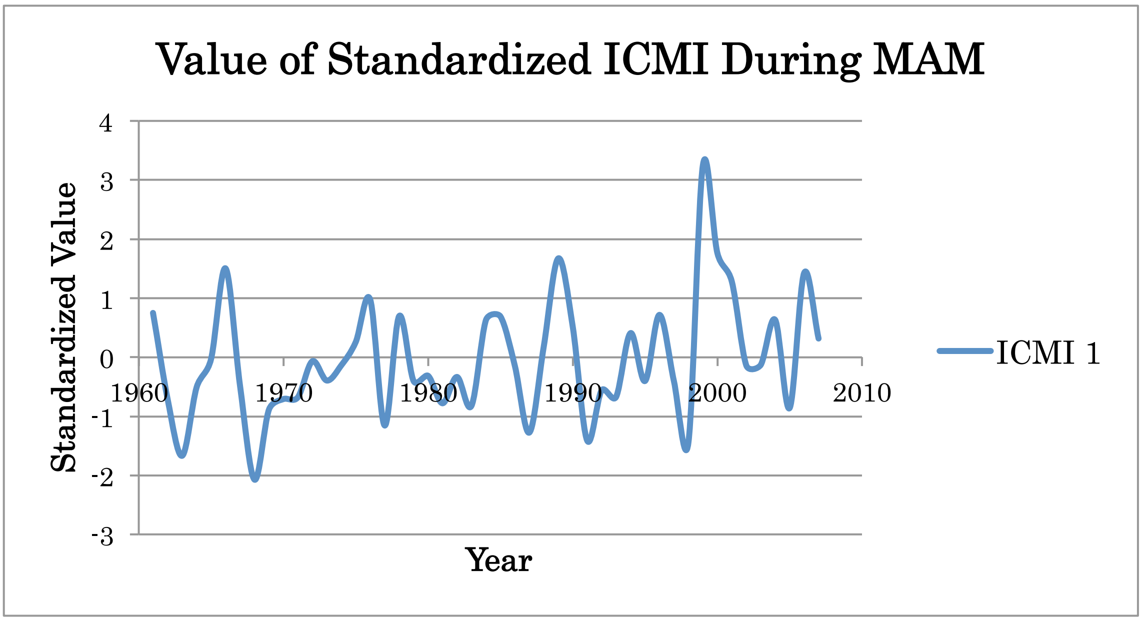
Value of Standardized ICMI During MAM
Standardized Value
4
3
2
1
0
-1
-2
-3
1960
1970
1980
1990
2000
2010
Year
ICMI 1

(b)

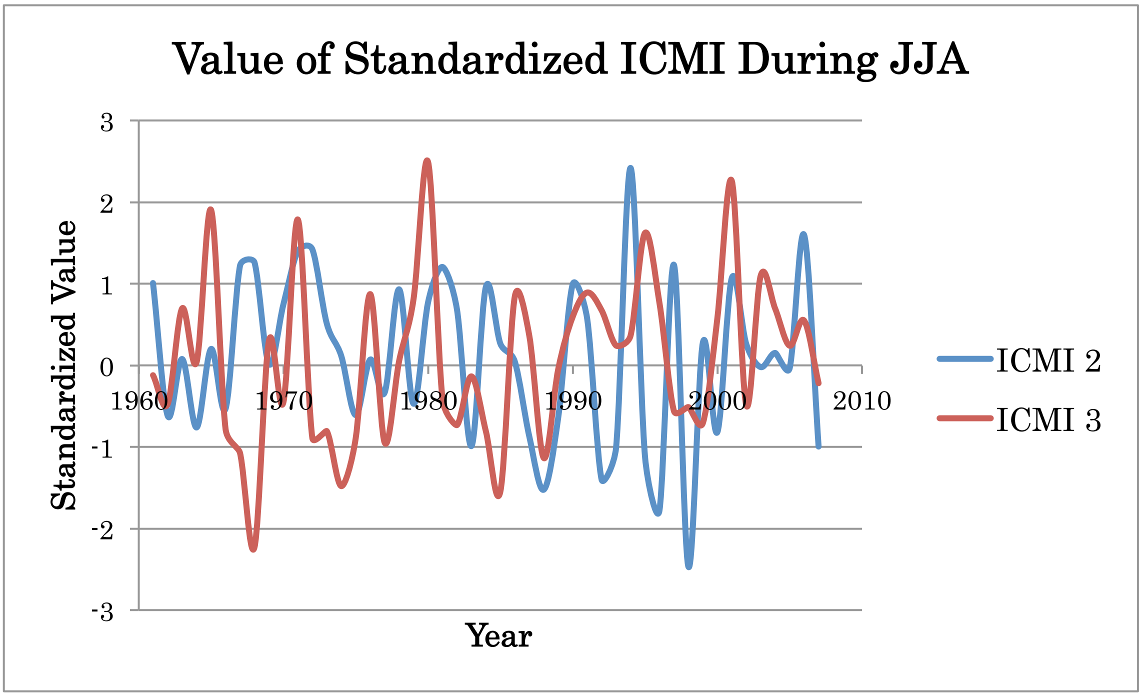
Value of Standardized ICMI During JJA
Standardized Value
3
2
1
0
-1
-2
-3
1960
1970
1980
1990
2000
2010
Year
ICMI 2
ICMI 3

(c)

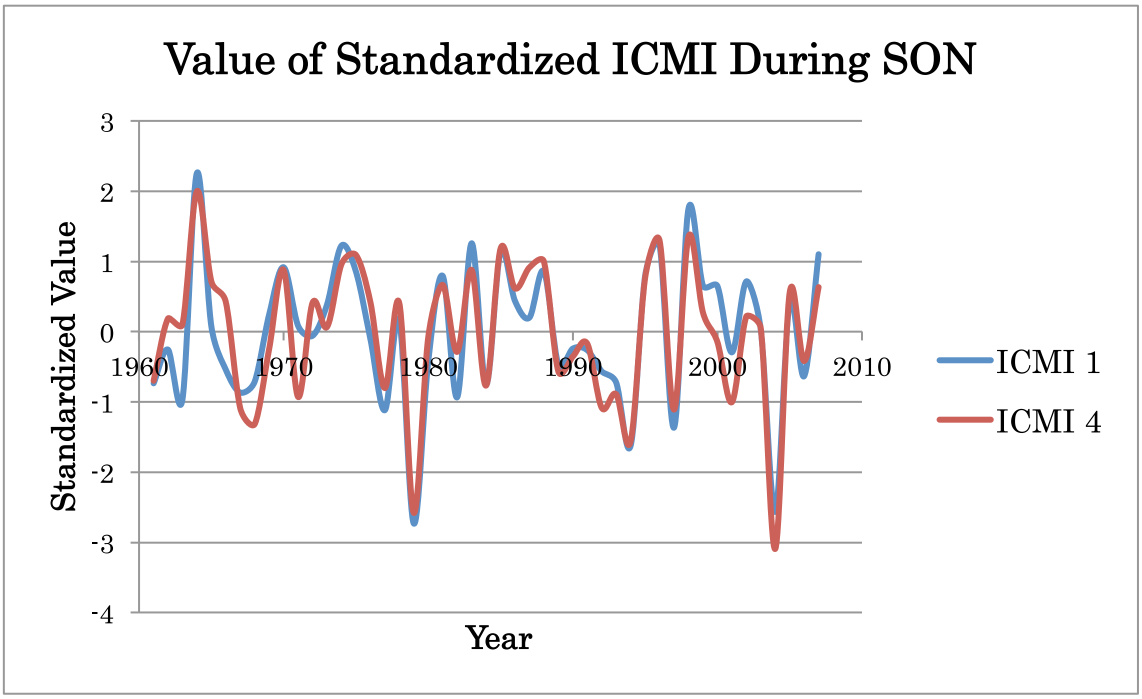

Figure 1: (a) Standardized Value of ICMI 1 during MAM 1961-2007. (b) Standardized Value of ICMI 2 and 3 during JJA 1961-2007. (c) Standardized Value of ICMI 1 and 4 during SON 1961-2007. This figure is generated by C.L.T. using Microsoft Excel.
